# Rescue of Enzymatic Function for Disease-associated RPE65 Proteins Containing Various Missense Mutations in Non-active Sites*[Fn FN2]

**DOI:** 10.1074/jbc.M114.552117

**Published:** 2014-05-21

**Authors:** Songhua Li, Tadahide Izumi, Jane Hu, Heather H. Jin, Ahmed-Abdul A. Siddiqui, Samuel G. Jacobson, Dean Bok, Minghao Jin

**Affiliations:** From the ‡ Department of Ophthalmology and Neuroscience Center, Louisiana State University Health Sciences Center, New Orleans, Louisiana 70112,; § Graduate Center for Toxicology, University of Kentucky College of Medicine, Lexington, Kentucky 40536,; ¶ Jules Stein Eye Institute and Department of Neurobiology, University of California, Los Angeles, California 90095,; ‖ Department of Biology, Washington University, St. Louis, Missouri 63130,; ** Department of Neuroscience, Tulane University, New Orleans, Louisiana 70118, and; ‡‡ Scheie Eye Institute, University of Pennsylvania, Philadelphia, Pennsylvania 19104

**Keywords:** Aggregation, Membrane, Photoreceptor, Proteasome, Retinal Degeneration, Retinoid, Ubiquitination, RPE65, PSMD13, Leber Congenital Amaurosis

## Abstract

Over 70 different missense mutations, including a dominant mutation, in RPE65 retinoid isomerase are associated with distinct forms of retinal degeneration; however, the disease mechanisms for most of these mutations have not been studied. Although some mutations have been shown to abolish enzyme activity, the molecular mechanisms leading to the loss of enzymatic function and retinal degeneration remain poorly understood. Here we show that the 26 S proteasome non-ATPase regulatory subunit 13 (PSMD13), a newly identified negative regulator of RPE65, plays a critical role in regulating pathogenicity of three mutations (L22P, T101I, and L408P) by mediating rapid degradation of mutated RPE65s via a ubiquitination- and proteasome-dependent non-lysosomal pathway. These mutant RPE65s were misfolded and formed aggregates or high molecular complexes via disulfide bonds. Interaction of PSMD13 with mutant RPE65s promoted degradation of misfolded but not properly folded mutant RPE65s. Many mutations, including L22P, T101I, and L408P, were mapped on non-active sites. Although their activities were very low, these mutant RPE65s were catalytically active and could be significantly rescued at low temperature, whereas mutant RPE65s with a distinct active site mutation could not be rescued under the same conditions. Sodium 4-phenylbutyrate and glycerol displayed a significant synergistic effect on the low temperature rescue of the mutant RPE65s by promoting proper folding, reducing aggregation, and increasing membrane association. Our results suggest that a low temperature eye mask and sodium 4-phenylbutyrate, a United States Food and Drug Administration-approved oral medicine, may provide a promising “protein repair therapy” that can enhance the efficacy of gene therapy by reducing the cytotoxic effect of misfolded mutant RPE65s.

## Teaser

**Background:** Disease-causing mutations reduce RPE65 protein levels with unknown mechanisms.

**Results:** Interaction of 26 S proteasome non-ATPase regulatory subunit 13 with mutant RPE65s mediates degradation of misfolded RPE65s via the ubiquitin-proteasome pathway.

**Conclusion:** Many mutant RPE65s with non-active site mutations are catalytically active and can be rescued.

**Significance:** Low temperature and chaperones that rescue enzymatic function of mutant RPE65s are therapeutic candidates.

## Introduction

Continuous regeneration of 11-*cis*-retinaldehyde, the universal light-sensing chromophore of the visual pigments in cone and rod photoreceptors, is essential for sustaining vision. RPE65, an abundant membrane-associated protein in the retinal pigment epithelium (RPE)^2^ (1, 2), is a key retinoid isomerase (3–5) necessary for regenerating 11-*cis*-retinaldehyde in the visual cycle. RPE65 utilizes hydrophobic all-*trans*-retinyl esters synthesized by lecithin:retinol acyltransferase (6) as substrate for the synthesis of 11-*cis*-retinol (3, 7), the reduced form of 11-*cis*-retinaldehyde. The RPE65-catalyzed isomerization reaction is a rate-limiting step in the visual cycle (8). Mice lacking RPE65 (*Rpe65*^−/−^ and rd12) cannot synthesize 11-*cis*-retinoids; therefore photoreceptors in these mice lose light sensitivity (9, 10).

Mutations in the human *RPE65* gene have been linked to retinal degenerative diseases such as Leber congenital amaurosis, retinitis pigmentosa, and childhood onset retinal dystrophy (11–15). So far, more than 100 different mutations have been identified in the *RPE65* gene of patients with the diseases (The Human Gene Mutation Database). Among these mutations, over 70 mutations are distinct missense mutations. Although most of these mutations have not been studied for their pathogenicity and disease mechanisms, 13 missense mutations tested severely eliminated retinoid isomerase activity of RPE65 (3, 5, 16–18). The enzyme activity measured in the laboratory was directly related to clinical effects of a mutation and can be used to distinguish pathogenic mutations from polymorphisms in the *RPE65* gene (5, 18).

The loss of RPE65 function involves at least two distinct mechanisms: loss of catalytic activity (3, 5) and a lower expression level of RPE65. Several missense mutations have been shown to cause rapid degradation of RPE65 in the kidney-derived HEK cell line (16–19) and mouse models (20–22). The molecular basis for the rapid degradation of mutant RPE65 is unknown. Understanding and pharmacological prevention of this rapid degradation may lead to the development of a therapeutic intervention.

Gene therapy that expresses wild-type RPE65 in patient's RPE can compensate for loss of RPE65 function and has provided some improvement of vision (23–28). However, a recent study showed that gene therapy could not stop retinal degeneration despite this visual improvement (29). In general, wild-type RPE65 expressed by gene therapy can confer enzyme activity to RPE, but it cannot stop the cytotoxic effect of mutated RPE65 if the mutant RPE65 has gained cytotoxic function. Recently, an autosomal dominant mutation in the *RPE65* gene has been found in patients with retinitis pigmentosa (30), suggesting that the mutated allele has a dominant pathogenic effect. Misfolding, mislocalization, and aggregation of mutant RPE65 (16, 17) may cause cytotoxic effects. Therefore, to enhance the gene therapy effect, it is important to develop an alternative strategy that can rescue the loss of function but also reduce cytotoxic function of mutated RPE65.

In this study, we investigated the common properties of several disease-causing RPE65s with regard to their molecular pathogenic mechanism and rescue of their enzyme activity. We found that the 26 S proteasome non-ATPase regulatory subunit 13 (PSMD13), a newly identified negative regulator of RPE65 (31), mediated degradation of misfolded mutant RPE65s through the ubiquitination-mediated proteasomal pathway in cultured human RPE cells. Many disease-causing RPE65s with a mutation in the non-active sites were catalytically active and could be significantly rescued by low temperature (30 °C) and chemical chaperone treatments.

## EXPERIMENTAL PROCEDURES

### 

#### 

##### Mouse Retinal Immunohistochemistry

All mouse experiments were approved by the Institutional Animal Care and Use Committee for the Louisiana State University Health Sciences Center and performed according to guidelines established by the Association for Research in Vision and Ophthalmology Statement for the Use of Animals in Ophthalmic and Vision Research. Retinal immunohistochemistry was carried out as described previously (32). In brief, retinal cryosections of 5-week-old 129S2/Sv mice (Charles River Laboratories) were incubated with primary antibodies at 4 °C overnight and with secondary antibodies at room temperature for 1 h. The primary antibodies were rabbit polyclonal antibody against PSMD13 (Proteintech Inc.) and mouse monoclonal antibody against RPE65 (Millipore). The secondary antibodies were Alexa Fluor 488- or Alexa Fluor 555-conjugated goat anti-rabbit and anti-mouse IgG (Invitrogen). Nuclei were labeled with DAPI. Fluorescence signals were captured with a Zeiss LSM 510 Meta laser confocal microscope with a 40× oil immersion objective.

##### Cell Culture and Transfection

The 293T-LC cells (3) were maintained in DMEM (Invitrogen) supplemented with 10% heat-inactivated fetal bovine serum (FBS) and antibiotics at 37 °C under 5% CO_2_. The ARPE-19 cells (33) were maintained in DMEM/F-12 supplemented with 10% FBS. Primary human RPE cells were established and maintained as described previously (34). For transfection, these human RPE cells were split into 24-well plates without Millicell-HA filters and incubated in modified Chee's medium (34) containing 5% FBS for 3 days. PolyJet (SignaGen Laboratories) or Lipofectamine 2000 (Invitrogen) was used for transfection. Relative transfection efficiencies were determined by measuring activities of β-gal from pRSV-LacZ (35) cotransfected with wild-type (WT) or mutant RPE65 constructs.

##### Low Temperature and Chemical Treatment of Cells

For low temperature treatment, cells were incubated at 30 °C under 5% CO_2_ for the indicated times. For chemical treatment, cells were incubated with the following reagents at the indicated final concentrations in the culture media. Proteasome inhibitors (9 μm MG132 and MG115, Sigma-Aldrich), 10 μm pepstatin A (Sigma-Aldrich), and a series of different concentrations of UBEI-41 (Biogenova) were incubated with cells for 16 h at 37 °C; 0–2 mm sodium 4-phenylbutyrate (PBA) and 0–0.4 m glycerol were incubated with cells for 30 h at 37 or 30 °C.

##### Site-directed Mutagenesis

PCR using a QuikChange II XL site-directed mutagenesis kit (Stratagene) was carried out to introduce a mutation into the pRK5-RPE65 plasmid encoding the human RPE65 (18). Primers used for generating mutant RPE65 constructs are shown in supplemental Table S1. Following mutagenesis, all sequences of the RPE65 coding region were confirmed by DNA sequence analysis.

##### Quantitative RT-PCR

Total RNA was extracted from the transfected 293T-LC cells using an RNA isolation kit with DNase I and reverse transcribed to cDNA using SuperScript III (Invitrogen). Quantitative PCR was performed on a C1000 Thermal Cycler (Bio-Rad) using the iQ SYBR Green Supermix (Bio-Rad) and 0.3 μm primer sets specific for the human RPE65 (5′-GAACTGTCCTCGCCGCTCAC and 5′-GCAGGGATCTGGGAAAGCAC) or GAPDH (5′-GGAAGGTGAAGGTCGGAGTCA and 5′-CTTCCCGTTCTCAGCCTTGAC). Each RPE65 construct was transfected into the cells in triplicate, and their mRNA levels were normalized to GAPDH mRNA levels. Relative content of RPE65 mRNA was calculated based on its threshold cycle (Ct) relative to that of GAPDH. The average mRNA levels of mutant RPE65 were compared with those of WT control.

##### Immunoblot Analysis

Proteins were separated in a 10 or 12% polyacrylamide gel by sodium dodecyl sulfate-polyacrylamide gel electrophoresis (SDS-PAGE) and transferred to an Immobilon-P membrane (Millipore). The membrane was incubated in blocking buffer, primary antibody, and secondary antibody as described previously (2). A polyclonal antibody against RPE65 peptide (corresponding to human RPE65 residues 150–164) was used to detect RPE65. No mutation site studied in this work is mapped on the peptide region. Immunoblots were visualized with ECL Prime and ImageQuant LAS4000 (GE Healthcare). The fluorescence intensity of each band was measured using ImageQuant TL software.

##### Immunoprecipitation

Cells expressing RPE65 and PSMD13-FLAG fusion protein were lysed in a 50 mm Tris-HCl buffer (pH 7.4) containing 150 mm NaCl, 1 mm EDTA, 0.5% Nonidet P-40, and protease inhibitors. Immunoprecipitation was carried out using an anti-FLAG M2 affinity gel (Sigma-Aldrich) as described previously (36). Precipitated proteins were used for immunoblot analysis in the presence of reducing reagents.

##### Knockdown of PSMD13

PSMD13 depletion in 293T-LC and ARPE-19 cells was performed by transfecting PSMD13 siRNA (OriGene Technologies, Inc.) using the PolyJet transfection reagent. Scrambled siRNA (OriGene Technologies, Inc.) was used as a negative control. The PSMD13 siRNA targets exon 9 of the human *PSMD13* gene (5′-GGAGATGACTTTCACACGACCTGCC-3′). Forty-eight hours post-transfection, the cells were used for immunoblot analysis.

##### His-Ubiquitin and Ubiquitin (Ub)-Agarose Pulldown Assays

Cells were transfected with plasmids encoding RPE65 or His_6_-ubiquitin fusion protein (His-Ub) (37). Proteins ubiquitinated with His-Ub were purified using Ni-NTA magnet beads (Qiagen) as described previously (37). In brief, 30 h after transfection, cells were incubated with 9 μm MG132 for 16 h and with 5 mm *N*-ethylmaleimide for 15 min at room temperature and harvested in ice-cold Ni-NTA buffer containing 20 mm Tris (pH 8.0), 500 mm NaCl, 0.05% Tween 20, 10 mm imidazole, 9 μm MG132, EDTA-free proteinase inhibitor mixture, and phenylmethylsulfonyl fluoride. After centrifugation, a small volume of the supernatant was taken as total cell lysate, and the rest was incubated with Ni-NTA magnet beads for 15 min at 4 °C. The beads were washed with the NTA buffer containing 10–20 mm imidazole and eluted with NTA buffer containing 200 mm imidazole. For the Ub-agarose pulldown assay, cell lysates in binding buffer (50 mm Tris (pH 7.5), 150 mm NaCl, 5 mm MgCl_2_, 5 mm KCl, and 1% Triton X-100) were incubated with 20 μl of Ub-agarose for 2 h at 4 °C. The beads were washed extensively with the buffer, and the bound proteins were analyzed by immunoblot analysis using an anti-E1 ubiquitin-activating enzyme (UBE1) antibody (Santa Cruz Biotechnology).

##### Retinoid Isomerase Assay

The 293T-LC cells were transfected with plasmid constructs encoding WT or mutant RPE65. Thirty hours after transfection, cells were incubated with 5 μm all-*trans*-retinol for 16 h at 30 or 37 °C. Retinoids were extracted from the cells and saponified as described previously (3).

##### Analysis of Retinoids

Retinoids were analyzed by normal-phase HPLC as described previously (38). In brief, retinoids in hexane extractions were evaporated, redissolved in 100 μl of hexane, and separated on a silica column (Zorbax-Sil, 5 μm, 250 × 4.6 mm, Agilent Technologies) by gradient (0.2–10% dioxane in hexane at 2.0 ml/min flow rate) or non-gradient (10% dioxane in hexane at 1.0 ml/min flow rate) elution of the mobile phase in an Agilent 1100 liquid chromatograph.

##### Analysis of Disulfide Bond-mediated High Molecular Weight Complexes (HMCs)

ARPE-19 cells expressing WT or mutant RPE65 were incubated with 15 μm E1 inhibitor (UBEI-41) for 16 h. After centrifugation at 1000 × *g* for 10 min, the cells were incubated in lysis buffer (PBS (pH 7.4), 0.1% SDS, and EDTA-free protease inhibitor mixture) containing or not containing reducing reagents (200 mm DTT and/or 2% 2-mercaptoethanol) for 30 min at room temperature or for 10 min at 70 °C. The reducing reagents promote reduction of cysteine disulfide bonds in proteins. The cell lysates were then subjected to immunoblot analysis.

##### Mapping of Mutation Sites onto the Crystal Structure of RPE65

The identity (code 4F2Z) of the crystal structure of bovine RPE65 in a lipid environment (39) was obtained from the RCSB Protein Data Bank. Mapping of mutation sites onto a three-dimensional structure of RPE65 was done using the program UCSF Chimera.

##### Immunocytochemistry

Cells grown on a glass coverslip were fixed with 4% paraformaldehyde and incubated with 0.2% Triton X-100 in PBS for 15 min. After blocking with 10% FBS and 2% goat serum in PBS for 1 h, the cells were incubated with RPE65 antibody overnight at 4 °C and then with Alexa Fluor 555-conjugated secondary antibody (Invitrogen) at room temperature for 1 h. Before and after incubating with antibodies, the cells were washed with 0.1% Tween 20 in PBS three times. Nuclei were labeled with DAPI, and images were captured with a Zeiss LSM 510 Meta confocal microscope.

##### Preparation of Cell Membrane Pellets

The 293T-LC cells expressing WT or mutant RPE65 were resuspended in ice-cold 10 mm HEPES buffer (pH 7.2) containing protease inhibitors and homogenized in a glass-to-glass tissue grinder. A portion of cell homogenates was centrifuged at 400 × *g* for 10 min. The resulting supernatants were centrifuged for 1 h at 100,000 × *g* at 4 °C to pellet the membranes. The membrane pellets were resuspended in the HEPES buffer containing 0.1% SDS and protease inhibitors. Proteins from the cell homogenates and the membrane fractions were analyzed by immunoblot analysis.

##### Statistical Analysis

Statistical significance was determined with an unpaired, two-tailed Student's *t* test. A *p* value less than 0.01 was considered to be statistically significant. Data are expressed as the mean ± S.D. of three or more independent experiments unless otherwise noted.

## RESULTS

### 

#### 

##### PSMD13 Promotes Degradation of Disease-causing RPE65s in Cultured Human RPE Cells

**FIGURE 1.**
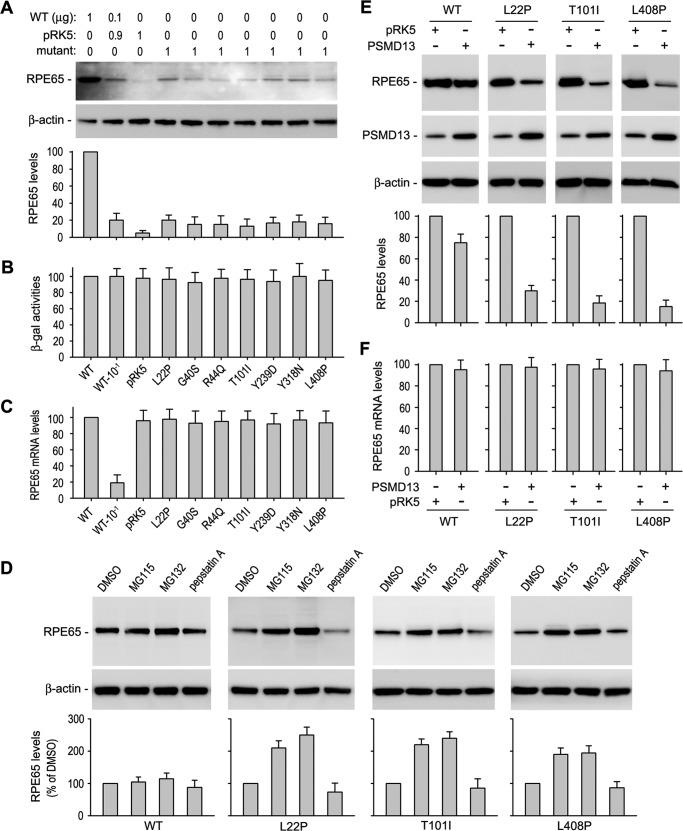
**PSMD13 promotes degradation of disease-causing RPE65s in the proteasome.** *A*, immunoblot analysis of cultured human RPE cells transfected with the indicated amount of constructs for WT RPE65, disease-associated RPE65s, or pRK5 mock vector. β-Actin was detected as a loading control. Relative intensities of the immunoblots were quantified using ImageQuant TL and expressed as a percentage of RPE65 in the cells transfected with 1 μg of WT RPE65. *B*, β-gal activities from pRSV-LacZ cotransfected with WT or the indicated mutant RPE65 into the human RPE cells. *C*, quantitative RT-PCR showing relative expression levels of mRNA for WT or mutant RPE65 in transfected human RPE cells. *D*, immunoblot analysis of ARPE-19 cells transfected with WT or the indicated mutant RPE65 constructs. The cells were treated with the indicated proteasome or lysosome inhibitors. Relative intensities of the immunoblots were quantified and expressed as a percentage of RPE65 in the DMSO-treated cells. *E*, immunoblot analysis of ARPE-19 cells expressing WT or the indicated mutant RPE65. The cells were cotransfected with pRK5 mock or PSMD13 constructs. Relative intensities of the immunoblots were quantified and expressed as a percentage of WT RPE65. *F*, quantitative RT-PCR showing relative expression levels of mRNA for WT or mutant RPE65 in ARPE-19 cells cotransfected with pRK5 or PSMD13 plasmid. All *error bars* show S.D. (*n* = 3).

We and others have shown that disease-causing missense mutations abolish RPE65 isomerase activity in the kidney-derived HEK cell line (3, 5, 16–18). To analyze the mechanism that results in loss of RPE65 function, we transiently expressed WT and disease-associated mutant (L22P, G40S, R44Q, T101I, Y239D, Y318N, and L408P) RPE65s in cultured human primary RPE cells, which retain many of the functional and morphologic characteristics of RPE *in vivo* (34, 40–42). To reduce the expression level of endogenous RPE65, we maintained the transfected cells in plastic culture plates instead of Millicell-HA chambers. As shown in Fig. 1*A*, the expression level of endogenous RPE65 in the cells transfected with pRK5 mock vector was ∼5% of total RPE65 (endogenous plus exogenous RPE65) in the cells transfected with WT RPE65 construct. Under the same experimental conditions, immunoblot analysis using a polyclonal antibody showed that expression levels of mutant RPE65s were less than 25% of WT RPE65 (Fig. 1*A*). We obtained a similar result with a monoclonal antibody against RPE65. The lower expression levels of mutant RPE65s were not due to lower transfection efficiency because activities of β-gal expressed from pRSV-LacZ cotransfected with WT and mutant RPE65 constructs were similar in all transfected cells (Fig. 1*B*). To confirm this result, we measured mRNA expression levels by quantitative RT-PCR. As shown in Fig. 1*C*, mRNA expression levels of mutant RPE65s were similar to that of WT RPE65.

To identify whether the proteasome or the lysosome is involved in the degradation of the mutant RPE65s, we treated cells with proteasome inhibitors (MG115 and MG132) or a lysosome inhibitor (pepstatin A). The content of mutant RPE65s in the cells treated with MG115 or MG132 was significantly increased compared with those in pepstatin A- or DMSO-treated cells (Fig. 1*D*), suggesting that the mutant RPE65s are mainly degraded in the proteasome.

We recently identified PSMD13 as a new negative regulator of RPE65 (31). Because PSMD13 is a positive regulator of the 26 S proteasome function (43), we tested whether PSMD13 promotes degradation of WT and the mutant RPE65s. We co-expressed PSMD13 with WT or mutant RPE65s in cultured human RPE cells. Quantitative immunoblot analysis showed that expression levels of mutant RPE65s (with an L22P, T101I, or L408P mutation) in pPSMD13-cotransfected cells were at least 50% lower than those in control cells cotransfected with pRK5 mock plasmid (Fig. 1*E*). The expression level of WT RPE65 was decreased only ∼15% in the pPSMD13-contransfected cells compared with the pRK5-contransfected cells (Fig. 1*E*). Quantitative RT-PCR showed that the mRNA expression levels of WT and mutant RPE65s were similar in all cotransfected cells (Fig. 1*F*), suggesting that PSMD13 promoted degradation of the mutant RPE65 proteins.

##### PSMD13 Interacts with Mutant RPE65s and Is an Essential Mediator for Degrading Mutant RPE65s

**FIGURE 2.**
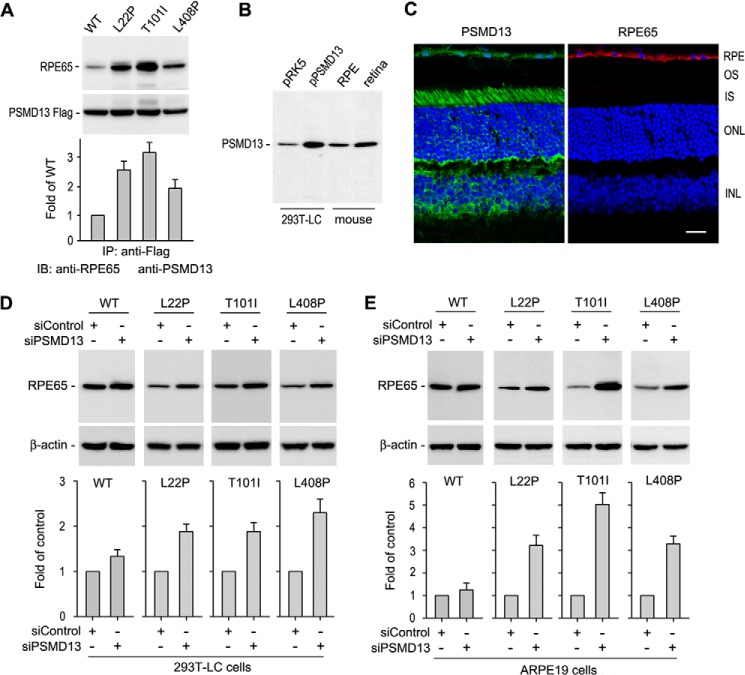
**PSMD13 mediates degradation of disease-causing RPE65s.** *A*, immunoprecipitation (*IP*) showing strong interaction of PSMD13 with the indicated mutant RPE65s. The 293T-LC cells expressing PSMD13-FLAG fusion protein and WT or mutant RPE65 were immunoprecipitated with a FLAG antibody, and the precipitates were probed with antibodies against RPE65 or FLAG epitope. Relative immunoblot (*IB*) intensities of RPE65 in the immunoprecipitates were normalized to the FLAG immunoprecipitates and expressed as -fold of WT RPE65. *B*, immunoblot analysis showing expression of PSMD13 in the mouse retina and RPE. 293T-LC cells transfected with pRK5 or pPSMD13 were used as controls. *C*, immunohistochemistry showing expression of PSMD13 and RPE65 in RPE. PSMD13 is also expressed in the photoreceptor inner segments (*IS*) and other types of retinal cells. *OS*, outer segment; *ONL*, outer nuclear layer; *INL*, inner nuclear layer. *D*, PSMD13 siRNA, but not nonspecific control siRNA, increased expression levels of the mutant RPE65s in 293T-LC cells. *E*, PSMD13 siRNA increased expression levels of the mutant RPE65s in ARPE-19 cells. All *error bars* denote S.D. (*n* = 3).

We next tested whether PSMD13 interacts with mutant RPE65s. We co-expressed PSMD13-FLAG with WT or mutant RPE65s in the 293T-LC cells. Immunoprecipitation using a FLAG antibody showed that PSMD13 interacted strongly with mutant RPE65s but weakly with WT RPE65 (Fig. 2*A*). Immunoblot analysis and immunohistochemistry revealed that PSMD13 is expressed in the mouse RPE and retina (Fig. 2, *B* and *C*).

If PSMD13 is an essential mediator for degrading mutant RPE65s, knockdown of PSMD13 should result in an increase in expression levels of mutant RPE65s. To test this possibility, we expressed WT and mutant RPE65s in 293T-LC cells transfected with PSMD13-specific or scrambled siRNA. The protein content of WT RPE65 in the cells cotransfected with the PSMD13 siRNA was increased 20% compared with that in the scrambled siRNA-cotransfected cells (Fig. 2*D*). In contrast, the contents of mutant RPE65s (with an L22P, T101I, or L408P mutation) were increased at least 100% in the PSMD13 siRNA-cotransfected cells compared with that in the nonspecific siRNA-cotransfected cells (Fig. 2*D*).

To confirm this result, we expressed WT and the mutant RPE65s in human RPE-derived ARPE-19 cells. Similar to the result observed in the 293T-LC cells, protein expression levels of the mutant RPE65s were significantly increased in the PSMD13 siRNA-cotransfected cells compared with the nonspecific siRNA-cotransfected cells (Fig. 2*E*), whereas the content of WT RPE65 was not significantly changed in the PSMD13-specific *versus* nonspecific siRNA-cotransfected cells (Fig. 2*E*), indicating that PSMD13 is an essential mediator for degrading mutant RPE65s.

##### Mutant RPE65s Are Strongly Ubiquitinated

**FIGURE 3.**
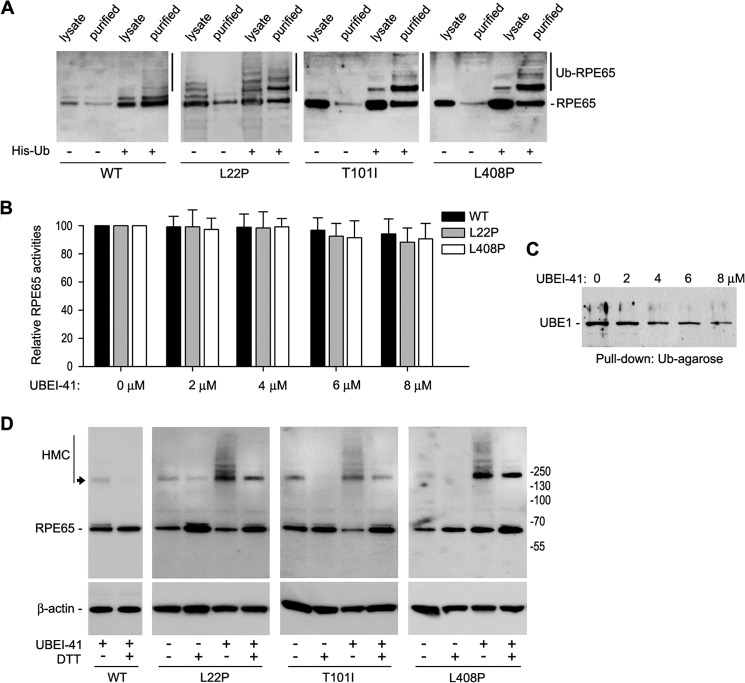
**Ubiquitination is required for degrading mutant RPE65s but not for regulating activity of RPE65.** *A*, ubiquitination of WT and mutant RPE65s in ARPE-19 cells. Cells expressing RPE65 and His-Ub were lysed and subjected to purification using Ni-NTA magnet beads. The cell lysates and purified fractions were probed with an RPE65 antibody. *B*, E1 enzyme inhibitor (UBEI-41) did not change isomerase activity of WT and mutant RPE65s. The 293T-LC cells expressing WT or mutant RPE65s were incubated with the indicated concentration of UBEI-41. Retinoid isomerase activities in these cells were expressed as a percentage of those in untreated cells. *Error bars* show S.D. (*n* = 3). *C*, UBEI-41 inhibited interaction of UBE1 with Ub. Cells lysates from *B* were incubated with Ub-agarose, and pulled down fractions were probed with an UBE1 antibody. *D*, inhibition of protein ubiquitination promotes HMC formation of mutant RPE65s via disulfide bonds. ARPE-19 cells expressing the indicated RPE65 were treated with UBEI-41 and subjected to immunoblot analysis in the presence or absence of reducing reagent (DTT). The bands indicated with an *arrow* may be tetramers of RPE65.

Ubiquitination has been shown to play an important role in protein degradation (44). To test whether WT and mutant RPE65s are ubiquitinated in the cells, we co-expressed His-Ub with WT or mutant RPE65 (L22P, T101I, or L408P) in ARPE-19 cells. Using Ni-NTA beads, we purified proteins ubiquitinated with His-Ub. Immunoblot analysis of the purified fractions and the total cell lysates demonstrated that mutant RPE65s were strongly ubiquitinated in the cells (Fig. 3*A*).

We next asked whether ubiquitination regulates enzyme activity of RPE65. To address this question, we treated 293T-LC cells expressing WT or mutant RPE65 with UBEI-41, an inhibitor of UBE1 (45). Because a high concentration of UBEI-41 was toxic to the 293T-LC cells, we measured retinoid isomerase activity in cells treated with 0–8 μm UBEI-41. As shown in Fig. 3*B*, relative enzyme activities of WT and mutant RPE65 in the cells treated with 2–8 μm UBEI-41 are similar to those in untreated cells. To confirm whether UBEI-41 inhibited UBE1 under these conditions, we performed a Ub-agarose pulldown assay. Immunoblot analysis of UBE1 in the Ub-agarose-precipitated fraction showed that UBEI-41 significantly inhibited interaction between UBE1 and Ub in the cells treated with 4–8 μm UBEI-41 (Fig. 3*C*).

##### Ubiquitination Is Important for Degradation of Misfolded Mutant RPE65s

It is known that ubiquitination mediates degradation of misfolded proteins (46). We therefore asked whether ubiquitination promotes degradation of misfolded mutant RPE65s. If ubiquitination is essential for degrading misfolded RPE65s, then inhibition of ubiquitination should result in accumulation of misfolded RPE65s. To test this possibility, we treated ARPE-19 cells expressing WT and mutant RPE65s with UBE1 inhibitor. As shown in Fig. 3*D*, HMCs containing mutant RPE65s accumulate significantly in UBE1 inhibitor-treated cells compared with DMSO-treated cells. These HMCs in the UBE1 inhibitor-treated cells were significantly reduced when the cell lysates were treated with DTT reducing reagent (Fig. 3*D*), suggesting that the mutant RPE65s are misfolded and formed HMCs via disulfide bonds.

##### Low Temperature Rescued Enzyme Activity of Disease-causing RPE65s with a Non-active Site Mutation but Not with an Active Site Mutation

**FIGURE 4.**
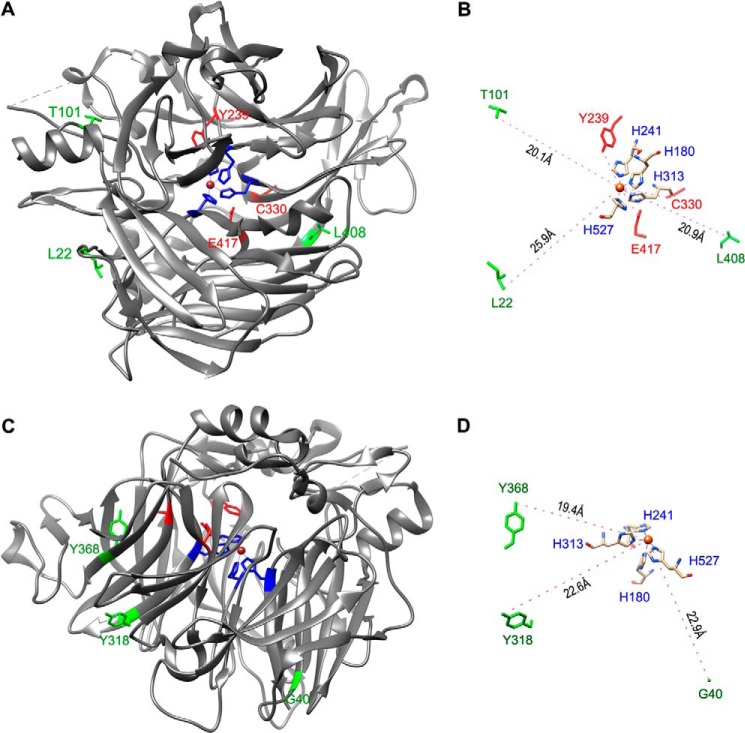
**Mapping of disease-causing mutation sites on the crystal structure of bovine RPE65.** *A*, a three-dimensional image of the crystal structure of bovine RPE65. The catalytic site containing Fe^2+^ (*brown sphere*) is in the center of RPE65. The three mutation sites (Leu-22, Thr-101, and Leu-408) shown in *green* are mapped in the non-active sites, whereas the other three mutation sites (Tyr-239, Cys-330, and Glu-417) shown in *red* are close to the active site cavity of RPE65. *B*, geometry differentiating the distances from the iron ion to the non-active site (*green*) and active site (*red*) mutations. Tyr-239, Cys-330, and Glu-417 are significantly closer to the iron ion with a separation of 6.9, 12.8, and 6.8Å, respectively. His-180, His-241, His-313, and His-527 are iron-binding histidines. *C* and *D*, three non-active site mutations (Gly-40, Tyr-318, and Tyr-368) are shown.

By mapping the three mutation sites (L22P, T101I, and L408P) onto the crystal structure of the bovine RPE65 (39, 47, 48), we found that these mutations are non-active site mutations (Fig. 4*A*). This observation prompted us to test whether low temperature, which has been shown to ameliorate proper folding of many other mutant proteins (36, 49, 50), can rescue enzyme activity of the mutant RPE65s. We incubated 293T-LC cells expressing mutant RPE65s at 30 °C and performed a retinoid isomerase assay at 30 °C. The isomerase activities of the three mutant RPE65s in these cells were increased at least 5-fold compared with their activities at 37 °C (Table 1). To confirm this result, we expressed three other disease-causing RPE65s (with a G40S, Y318N, or Y368H mutation) in the cells. These three mutations are also non-active site mutations (Fig. 4, *C* and *D*). Isomerase assays showed that activities of these mutant RPE65s were increased ∼3-fold compared with their activities at 37 °C (Table 1). These results suggest that isomerase activities of mutant RPE65 at 30 and 37 °C may provide a useful parameter to predict whether a mutation is an active site mutation or non-active site mutation.

**TABLE 1:** **Rescue of disease-causing non-active site mutant RPE65s at low temperature**

Mutation	Activity at 30 °C*^a^*	Activity at 37 °C*^a^*	Ratio (30 °C/37 °C)*^b^*
L22P	22.4 ± 2.6	3.9 ± 0.5	5.8
T101I	12.6 ± 1.7	2.4 ± 0.8	5.3
L408P	25.9 ± 2.7	5.0 ± 0.8	5.2
G40S	3.9 ± 0.5	1.3 ± 0.3	3.0
Y318N	24.8 ± 2.3	7.1 ± 1.2	3.5
Y368H	3.6 ± 0.9	1.2 ± 0.3	3.0
WT	124 ± 10	138 ± 12	0.9

*^a^* Retinoid isomerase activities of WT and the disease-causing mutant RPE65s were determined by measuring synthesis of 11-*cis*-retinol in the 293T-LC cells incubated with all-*trans*-retinol at 30 or 37 °C. Numbers indicate 11-*cis*-retinol content (in pmol; ±S.D.) in 1 mg of cellular protein (*n* = 3).

*^b^* Ratio of the retinoid isomerase activities at 30 °C to those at 37 °C.

To test this possibility, we substituted the conserved iron-binding histidine residues (5, 51) with arginine individually. These mutant RPE65s (with an H180R, H241R, H313R, or H527R substitution) were then expressed in 293T-LC cells at 30 or 37 °C. Retinoid isomerase assays showed that these mutant RPE65s had no activity at 30 and 37 °C (Table 2), suggesting that low temperature could not rescue active site mutant RPE65s. To confirm this result in disease-causing mutations, we generated mutant RPE65s with an Y239D, C330Y, or E417Q mutation. These mutation sites are close to the catalytic site (Fig. 4*B*). We then expressed these mutant RPE65s in the cells at 30 or 37 °C. The isomerase assays showed that low temperature could not rescue the enzyme activity of these mutant RPE65s (Table 2).

**TABLE 2:** **Low temperature could not rescue the indicated RPE65s with a distinct active site mutation**

Mutation	Activity at 30 °C*^a^*	Activity at 37 °C*^a^*	Ratio (30 °C/37 °C)*^b^*
H180R	ND*^c^*	ND	
H241R	ND	ND	
H313R*^d^*	ND	ND	
H527R	ND	ND	
Y239D*^d^*	1.3 ± 0.3	1.3 ± 0.5	1.0
C330Y*^d^*	1.8 ± 0.3	1.6 ± 0.3	1.1
E417Q*^d^*	1.2 ± 0.4	1.1 ± 0.4	1.1

*^a^* Retinoid isomerase activities of the indicated mutant RPE65s were determined by measuring synthesis of 11-*cis*-retinol in the 293T-LC cells incubated with all-*trans*-retinol at 30 or 37 °C. Numbers indicate 11-*cis*-retinol content (in pmol; ±S.D.) in 1 mg of cellular protein (*n* = 3).

*^b^* Ratio of the retinoid isomerase activities at 30 °C to those at 37 °C.

*^c^* ND, no detectable enzyme activity.

*^d^* Disease-associated mutations.

##### PSMD13 Had a Reduced Effect on Degradation of Non-active Site Mutant RPE65s at Low Temperature

**FIGURE 5.**
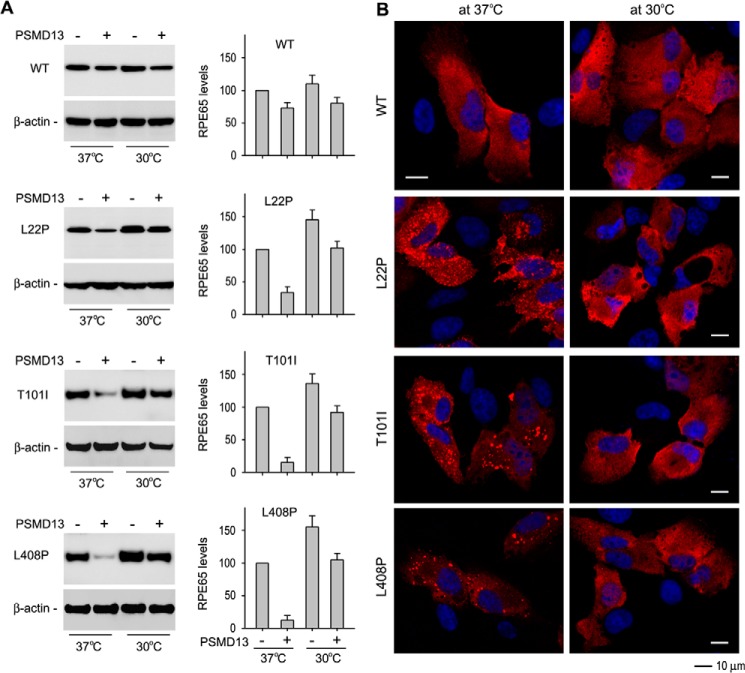
**Low temperature increased expression levels but inhibited aggregation of mutant RPE65s.** *A*, low temperature increased expression levels of mutant RPE65s by reducing PSMD13-mediated degradation. ARPE-19 cells cotransfected with the mutant RPE65 and pPSMD13 or pRK5 were grown at 37 or 30 °C and subjected to immunoblot analysis using antibodies against RPE65 or β-actin. *Error bars* show S.D. (*n* = 3). *B*, low temperature prevented aggregation of mutant RPE65s. ARPE-19 cells transfected with WT or the indicated mutant RPE65 plasmid were incubated at 37 or 30 °C, stained with RPE65 antibody, and observed using a confocal microscope. *Scale bars* denote 10 μm.

The results described above suggest that low temperature can rescue non-active site mutant RPE65s by promoting proper folding of the mutant RPE65s. If PSMD13 mediates degradation of misfolded mutant RPE65, then PSMD13 should exhibit a reduced effect on degradation of mutant RPE65s at low temperature. We tested this possibility by co-expressing mutant RPE65s (L22P, T101I, or L408P) with PSMD13 in ARPE-19 cells at 30 °C and 37 °C. As shown in Fig. 5*A*, co-expression of PSMD13 significantly reduced expression levels of mutant RPE65s at 37 °C. In contrast, expression levels of mutant RPE65s at 30 °C were significantly increased in both cells co-expressing and those not co-expressing PSMD13 (Fig. 5*A*), suggesting that PSMD13 mediated degradation of misfolded mutant RPE65s and that low temperature ameliorated proper folding of the mutant RPE65s.

##### Low Temperature Inhibits Aggregate Formation of the Non-active Site Mutant RPE65s

Because mutant RPE65s are misfolded at 37 °C, we hypothesized that the mutant RPE65s form aggregates in the cells. To test this possibility, we expressed the three non-active site mutant RPE65s in ARPE-19 cells at 37 °C and then performed immunocytochemistry. As predicted, confocal microscopy showed that the mutant RPE65s formed numerous aggregates in the cells (Fig. 5*B*). To test whether low temperature can prevent or reduce the formation of mutant RPE65 aggregates, we performed the same experiment on cells grown at 30 °C. Immunocytochemistry showed that aggregate formation of the mutant RPE65s was significantly reduced at 30 °C (Fig. 5*B*).

##### Chemical Chaperones Enhanced Low Temperature Rescue of the Non-active Site Mutant RPE65s

**FIGURE 6.**
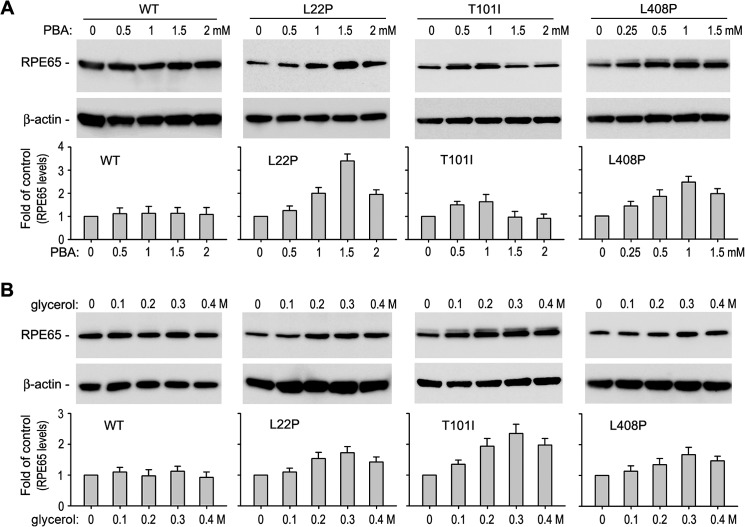
**Chemical chaperones increased expression levels of disease-associated RPE65s.** ARPE-19 cells transfected with WT or mutant RPE65 constructs were treated with the indicated concentration of PBA (*A*) or glycerol (*B*) and analyzed by immunoblot analysis using antibodies against RPE65 or β-actin. Histograms show relative expression levels of RPE65. All *error bars* show S.D. (*n* = 3).

The rescue of enzyme activity of the mutant RPE65s at 30 °C suggests that chemical chaperones that have been shown to help proper folding of other retinal mutant proteins (36, 52–55) can also rescue the non-active site mutant RPE65s. We therefore first tested the effect of PBA or glycerol on expression levels of WT and mutant RPE65s in ARPE-19 cells. As show in Fig. 6, expression levels of WT RPE65 were not significantly changed by treatment with PBA or glycerol, whereas expression levels of the three mutant RPE65s were significantly increased in the cells treated with 1 mm PBA or 0.3 m glycerol (Fig. 6).

**FIGURE 7.**
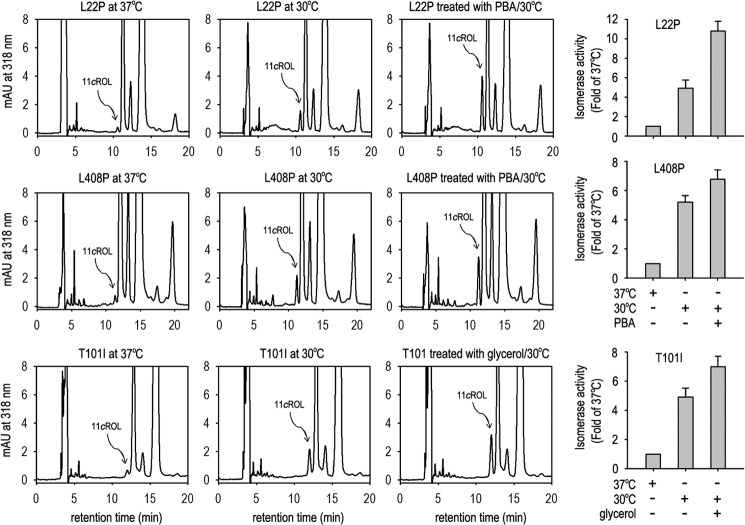
**Chemical chaperones enhanced low temperature-mediated rescue of enzyme activity of mutant RPE65s.** Shown are chromatograms of retinoids from 293T-LC cells expressing L22P, T101I, or L408P RPE65 at 37 or 30 °C in the presence or absence of PBA or glycerol. The peak of 11-*cis* retinol (*11cROL*) is marked by an *arrow*. The histograms show relative enzyme activity of the mutant RPE65s in the indicated conditions. All *error bars* show S.D. (*n* = 3). *mAU*, milli-absorbance units.

We next tested whether PBA and glycerol can enhance the low temperature-mediated rescue of enzyme activity of mutant RPE65s. We expressed the three mutant RPE65s in cells at 37 °C, at 30 °C, and at 30 °C with 1 mm PBA or 0.3 m glycerol. Retinoid isomerase assays in these cells showed that the enzyme activities of the three mutant RPE65s at 30 °C in the presence of PBA or glycerol were increased at least 30% or 7-fold compared with their activities at 30 °C in the absence of the chemical chaperone or at 37 °C (Fig. 7), respectively.

##### Chemical Chaperones and Low Temperature Promoted Association of Mutant RPE65s with Membranes

**FIGURE 8.**
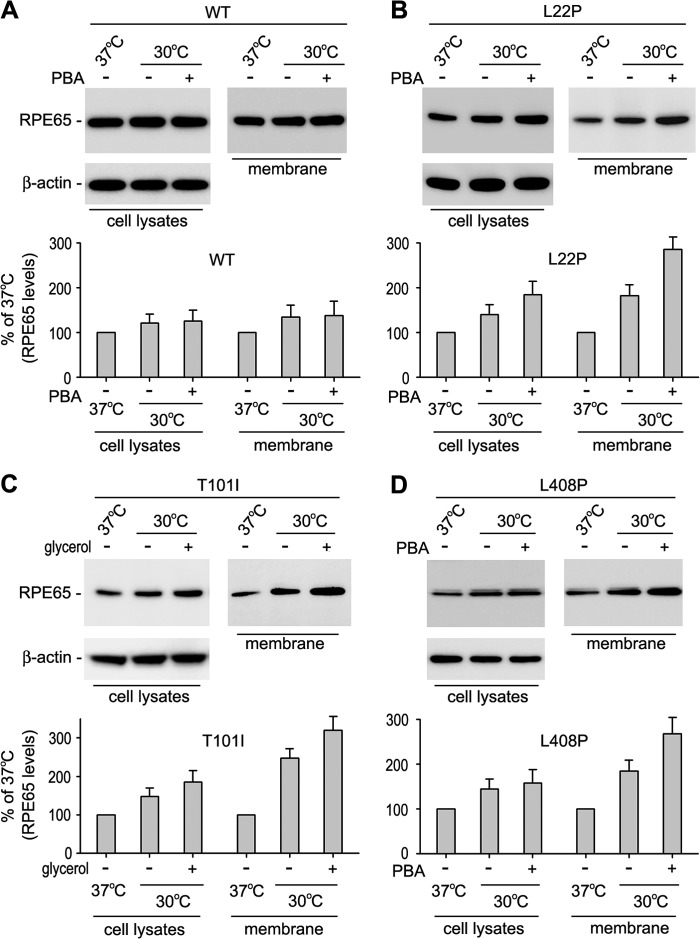
**PBA and low temperature synergistically increased association of mutant RPE65s with membranes.** Cells expressing the indicated mutant RPE65 were maintained at 37 or 30 °C in the presence or absence of PBA or glycerol. Total cell lysates and membrane fractions were analyzed by immunoblot analysis using antibodies against RPE65 or β-actin. Histograms show relative expression levels of the mutant RPE65 in PBA- or glycerol-treated *versus* untreated cells. *Error bars* show S.D. (*n* = 3).

It has been shown that association of RPE65 with membranes is important for its function (2, 48, 56, 57). Chemical chaperones and low temperature might enhance association of the mutant RPE65s with membranes. To test this possibility, we analyzed the relative content of WT and the three mutant RPE65s (L22P, T101I, and L408P) in membrane fractions and total homogenates of 293T-LC cells grown at 37 °C, at 30 °C, or at 30 °C in the presence of 1 mm PBA or 0.3 m glycerol. All mutant RPE65s were increased 40–48 or 58–85% in total homogenates of the cells maintained at 30 °C in the absence or presence of chemical chaperone, respectively, compared with the total homogenates of the cells maintained at 37 °C (Fig. 8). In contrast, the mutant RPE65s were increased 82–140 or 168–220% in the membrane fractions of the cells maintained at 30 °C in the absence or presence of chemical chaperones, respectively, compared with the membrane fractions of the cells maintained at 37 °C (Fig. 8).

## DISCUSSION

Previous studies by us and others have shown that some disease-associated RPE65s display significantly lower expression levels in HEK cell line (16–19). Similar results have also been seen in mouse models having an R91W or F229S mutation in the *Rpe65* gene. The steady-state contents of RPE65 in mouse RPE were reduced by 85–95% (20–22). In this study, we showed that expression levels of the seven distinct disease-causing RPE65s were at least 75% less than that of WT RPE65 in cultured primary human RPE cells (Fig. 1*A*).

Recently, Wright *et al.* (58) reported that mRNA encoding the mouse rd12-associated RPE65 could not bind normally to the ribosome for translation. Although we did not determine binding efficiency between the ribosome and mRNAs encoding the human mutant RPE65s, the following results suggest that the lower expression levels of the human mutant RPE65 proteins are mainly due to proteasomal degradation. 1) PSMD13, which positively regulates proteasome function (43), significantly reduced protein levels of the mutant RPE65s (Fig. 1*E*). 2) Knockdown of PSMD13 significantly increased expression levels of the mutant proteins (Fig. 2, *D* and *E*). 3) Proteasome inhibitors (MG115 and MG132), but not the lysosome inhibitor (pepstatin A), significantly rescued protein expression levels of the mutant RPE65s (Fig. 1*D*). 4) The mutant RPE65s were ubiquitinated in the cells (Fig. 3*A*).

Protein ubiquitination plays an important role in protein quality control by mediating degradation of misfolded proteins in the proteasome (46). For efficient targeting of proteins to the proteasome, multiple ubiquitins are often conjugated to the protein to form polyubiquitin chains (46, 59). We observed mono- and polyubiquitinated mutant RPE65s in transfected ARPE-19 cells (Fig. 3*A*). This observation and the significant increase of mutant RPE65s in the proteasome inhibitor-treated cells (Fig. 1*D*) suggest that the ubiquitin-proteasome pathway plays a critical role in degradation of mutant RPE65s. We also detected a relatively small amount of ubiquitinated WT RPE65 (Fig. 3*A*). These data are in agreement with the mild effect of proteasome inhibitors on the expression level of WT RPE65 (Fig. 1*D*).

The role of PSMD13 in vision and retinal health remains poorly understood. In our previous study attempting to identify an inhibitor(s) of RPE65 in RPE, we isolated the PSMD13 clone as a candidate for RPE65 inhibitor (31). RPE65 activity was reduced ∼25% in pPSMD13-transfected cells (31). This might be due to the slight promotion of WT RPE65 degradation by PSMD13 co-expression (Fig. 1*E*). Abundant expression of PSMD13 in mouse RPE and retina (Fig. 2, *B* and *C*) suggests that PSMD13 could regulate RPE65 activity by regulating degradation of RPE65. More importantly, PSMD13 strongly promoted degradation of the disease-causing RPE65s. Knockdown of PSMD13 significantly reduced degradation of mutant RPE65s (Fig. 2, *D* and *E*), indicating that PSMD13 is an essential mediator for degrading the mutant RPE65s. The significant reduction of the PSMD13 effect on degradation of the mutant RPE65s at low temperature (Fig. 5*A*) suggests that PSMD13 mediates degradation of mutant RPE65s that are misfolded at 37 °C (Figs. 3*D* and 5*B*). This may be a beneficial role of PSMD13 because it can reduce accumulation of misfolded cytotoxic RPE65 in the RPE. Conversely, PSMD13 may also play a harmful role by promoting degradation of non-active site mutant RPE65s that are misfolded but possess low enzyme activity (Table 1). In any case, PSMD13 plays a critical role in regulation of pathogenicity of mutant RPE65s and provides a therapeutic target.

Recent identification of a dominant pathogenic mutation in RPE65 (30) has increased the complexity of pathogenic mechanisms for RPE65 mutations. Many mutations, including recessive mutations, may result in both loss of function and gain of cytotoxic function due to misfolding and accumulation of mutated protein. Although the mutations analyzed in this work are recessive mutations, the mutated RPE65s formed HMCs via disulfide bonds (Fig. 3*D*). The intermolecular complexes may contain not only mutant RPE65s but also other proteins because the HMCs did not form clear ladders (Fig. 3*D*). These HMCs may also be the structural basis for the RPE65 aggregates observed in the cells (Fig. 5*B*). When PSMD13-mediated degradation of misfolded RPE65s is functional, mutant RPE65s cannot cause a severe cytotoxic effect. However, when misfolded RPE65s accumulate for some reason, they may become a chronic cytotoxic factor. This possibility is supported by recent studies. 1) Proteasome overload has been identified as a common stress factor in multiple forms of inherited retinal degeneration (60). 2) Human *RPE65* gene therapy could not stop retinal degeneration caused by recessive RPE65 mutations despite the fact that gene therapy improved visual function in the patients (29). Although the cellular and molecular mechanisms for the latter case need to be elucidated, it is clear that eliminating misfolded proteins in the RPE is important for retinal health.

Low temperature has been shown to reduce cellular damage by promoting proper folding of many mutated proteins (36, 49, 50). Glaucoma-causing mutant myocilin and retinitis pigmentosa-associated interphotoreceptor retinoid-binding protein cause endoplasmic reticulum stress due to misfolding and subsequent accumulation in the endoplasmic reticulum (36, 61). At low temperature, these mutant proteins can be secreted, and endoplasmic reticulum stress is reduced (36, 50). Similarly, we observed that low temperature significantly rescued the enzymatic activity of all six distinct disease-causing mutant RPE65s (Table 1). These results suggest that these mutant RPE65s are catalytically active. In support of this possibility, we found that their mutation sites are localized on the surface of the crystal structure of the bovine RPE65 (Fig. 4), indicating that the six mutations are non-active site mutations. Although the biochemical attribute of amino acid residues mutated is important in determining the enzyme activity of a mutant RPE65 (19), our result also suggests that the relative spatial distance between a mutation site and the catalytic site is a critical factor in determining whether the mutant RPE65 can be rescued (Fig. 4 and Tables 1 and 2). Importantly, many disease-associated missense mutations in RPE65 are non-active site mutations. Further studies are needed to confirm whether these mutations can also be rescued at low temperature.

To test whether active site mutations can be rescued at low temperature, we substituted the four iron-binding histidine residues with arginine individually. Both His and Arg are positively charged amino acids. Retinoid isomerase assays showed that these four mutant RPE65s had virtually no activity at 37 and 30 °C (Table 2). Consistent with these results, three disease-causing RPE65s whose mutation sites (Y239D, C330Y, and E417Q) are close to the catalytic site cavity (Fig. 4) could not be rescued at low temperature (Table 2). These results suggest that the mechanism resulting in loss of enzyme activity of the mutant RPE65s with an active site mutation is different from that of the mutant RPE65s with a non-active site mutation. The results also suggest that identifying whether a mutation is at the catalytic site cavity is important for determining whether physical and chemical approaches can be applied to rescue the mutant RPE65.

Chemical chaperones such as PBA and glycerol have been shown to reverse cellular mislocalization and rescue the function of many other mutated proteins (36, 52–54). These chemical chaperones and low temperature exhibited a significant synergistic effect on rescue of the non-active site mutant RPE65s (Fig. 7) by helping proper folding, reducing aggregation, increasing expression levels, and enhancing membrane association of the mutant RPE65s (Figs. 5–8). These results suggest that low temperature and chemical chaperones not only can restore normal function to the mutant RPE65s but also can reduce the cytotoxic effect of the mutant RPE65s. PBA is a United States Food and Drug Administration-approved safe oral medication (52, 62). Recently, PBA was shown to prevent disease phenotypes in a mouse model of primary open angle glaucoma caused by a mutation in the myocilin gene (54).

Recent gene therapy for patients with RPE65 mutations has shown safety and visual improvement (23–28). However, continuing retinal degeneration in patients who received *RPE65* gene therapy (29) suggests that a combinational therapy may be needed to improve vision and to prevent retinal degeneration. A low temperature eye mask and PBA functioning as a “protein repair therapy” (63) may be promising candidates for the combinational therapy.

## Supplementary Material

Supplemental Data

## Footnotes

2 The abbreviations used are:

RPE: retinal pigment epithelium
PSMD13: 26 S proteasome non-ATPase regulatory subunit 13
PBA: sodium 4-phenylbutyrate
Ub: ubiquitin
Ni-NTA: nickel-nitrilotriacetic acid
HMC: high molecular weight complex
UBE1: E1 ubiquitin-activating enzyme.
