# Supplementary material for: Rescue of Enzymatic Function for Disease-associated RPE65 Proteins Containing Various Missense Mutations in Non-active Sites
Source: J Biol Chem. 2014 May 21;289(27):18943–56. doi: 10.1074/jbc.M114.552117 (PMC4081934; doi:10.1074/jbc.M114.552117)
Supplement: Supplemental Data [file supp_289_27_18943__index.html]

Rescue of enzymatic function for disease-associated RPE65s containing various missense mutations in non-active sites — Rescue of Enzymatic Function for Disease-associated RPE65 Proteins Containing Various Missense Mutations in Non-active Sites — Rescue of Isomerase Activity for Disease-associated RPE65s — Supplemental Data 

# Rescue of Enzymatic Function for Disease-associated RPE65 Proteins Containing Various Missense Mutations in Non-active Sites

## Supplemental Data

**Files in this Data Supplement:**

- JBC\_2014\_552117\_S Li et al (.pdf, 90 KB) - Table S1. Primers used for generating the indicated mutant RPE65s
