# Supplementary material for: Rescue of Enzymatic Function for Disease-associated RPE65 Proteins Containing Various Missense Mutations in Non-active Sites
Source: J Biol Chem. 2014 May 21;289(27):18943–56. doi: 10.1074/jbc.M114.552117 (PMC4081934; doi:10.1074/jbc.M114.552117)
Supplement: Supplemental Data [file supp_M114.552117_jbc.M114.552117-1.pdf]

Supplemental Information

Table S1. Primers used for generating the indicated mutant RPE65s

Mutation	Forward primers (5' – 3')	Reverse primers (5' – 3')
L22P	gtttgaaactgtggaggaaccgtcctcgccgctcac	gtgagcggcgaggacggttcctccacagtttcaaac
G40S	cccctctggctcaccagcagtctccttcgatgtg	cacatcgaaggagactgctggtgagccagagggg
R44Q	caccggcagtctccttcaatgtgggccaggactc	gagtcctggcccacattgaaggagactgccggtg
T101I	gaaaaggatcgtcataatagaatttggcacctgtg	cacaggtgccaaattctattatgacgatccttttc
H180R	caatggggccactgctcgcccccacattgaaaatg	cattttcaatgtgggggcgagcagtggccccattg
Y239D	ccgattcaagccatctgacgttcatagttttggtctg	cagaccaaaactatgaacgtcagatggcttgaatcgg
H241R	caagccatcttacgttcgtagttttggtctgactcc	ggagtcagaccaaaactacgaacgtaagatggcttg
H313R	ctcctttcaacctcttccgtcacatcaacacctatgaag	cttcataggtgttgatgtgacggaagaggttgaaaggag
Y318N	cttccatcacatcaacaccaatgaagacaatgggtttctg	cagaaacccattgtcttcattggtgttgatgtgatggaag
C330Y	ctgattgtggatctctgctactggaaaggatttgagtttg	caaactcaaatcctttccagtagcagagatccacaatcag
Y368H	ccaacctgaagttaggagacatgtacttcctttgaatattg	caatattcaaaggaagtacatgtctcctaacttcaggttgg
L408P	gctggagcctgaagttcccttttcagggcctcg	cgaggccctgaaaagggaacttcaggctccagc
E417Q	gcctcgtcaagcatttcagtttcctcaaatcaattacc	ggtaattgatttgaggaaactgaaatgcttgacgaggc
H527R	catccctgtcacctttcgtggactgttcaaaaaatcttg	caagattttttgaacagtccacgaaaggtgacagggatg
